# Evaluation of a pregnancy programme to enhance older primiparas' physical and mental health and marital relationships after childbirth: A non‐randomized clinical trial

**DOI:** 10.1002/nop2.1746

**Published:** 2023-05-11

**Authors:** Kumiko Nakajima, Ayano Hirose, Tomoko Nameda

**Affiliations:** ^1^ School of Nursing, Faculty of Nursing Gunma Paz University Takasaki Gunma Japan; ^2^ School of Nursing, Graduate School of Nursing Gunma Prefectural College of Health Sciences Maebashi Japan

**Keywords:** marital relationship, non‐randomized clinical trial, older primiparas, physical and mental health, programme evaluation

## Abstract

**Aim:**

This study evaluated a pregnancy programme designed by us to stabilize older primiparas' physical and mental health and strengthen their marital relationships.

**Design:**

A non‐randomized controlled trial study of two groups; an intervention and control group.

**Methods:**

Ultimately the scores of 15 participants assigned to an intervention group and 15 assigned to a control group were analysed. Participants responded to sociodemographic questions, the Edinburgh Postnatal Depression Scale (EPDS), a postpartum physical fatigue questionnaire, wives' satisfaction with husbands' support questionnaire and Quality Marriage Index (QMI). Data were collected during pregnancy and at one and 3 months after childbirth.

**Results:**

The participating wives' EPDS scales significantly decreased after the postpartum course in the intervention group. Participating in the programme significantly raised husbands' awareness of their wives' physical burdens 1 month after childbirth. The subscale ‘housework support/wives' satisfaction with husbands' support’, 3 months after childbirth, did not decline. It is suggested that this programme could strengthen marital relationships because the husbands' understanding of their spouses' physical burdens after childbirth led to an improvement in the wives' satisfaction with their spouses' housework support. Participation in the pregnancy programme may strengthen the marital relationship. This study recommends appropriate nursing support for pregnant couples to improve their physical and mental health.

## 1 INTRODUCTION

The proportion of older primiparas in Japan's total number of births has nearly tripled over the past 20 years, from 11.9% in 2000 to 29.1% in 2019 (Ministry of Health, Labor, and Welfare of Japan, 2019). Older primiparas (over the age of 35) have a higher incidence of pregnancy complications and childbirth abnormalities (Japan Association of Obstetricians and Gynaecologists). In addition, older primiparas are more likely to suffer from child‐rearing anxiety and depression compared to younger primiparas and multiparous women (Sakajo et al., 2014; Satoh et al., 2009). For couples who become parents for the first time, adapting to the parental and changed social role is difficult. In Japan, there is a tradition of ‘satogaeri’ (wife's homebirth), and about 60% of primiparas receive postpartum care from their parents for the first month after childbirth. Newly parenting couples are thus more likely to rely on their wives' parents during the first month postpartum, making it more difficult for the couple to begin building their relationship during that time (Boda et al., 2020). In addition, uniquely among developed countries, husbands in Japan spend only 1 h and 54 min doing housework and childcare, which is shorter than wives' 7 h and 28 min (Ministry of Internal Affairs and Communications, 2021). The lack of support for housework and childcare by husbands and postpartum depression and crises resulting from mothers' solitary child‐rearing have become societal problems. When a couple has children, the wife experiences an increased burden of housework and childcare. If the husband does not understand the wife's physical and emotional burdens, she may become frustrated at the lack of sufficient cooperation from her spouse. Their marital relationship may suffer therefore (Belsky et al., 1995), and a postpartum crisis is likely to occur (Uchida & Tsuboi, 2013). Parenting for the first time presents couples with a developmental crisis that affects the couple's relationship, therefore anticipatory guidance is extremely important (Suto et al., 2017). It is necessary to provide a programme that addresses the relationship between husbands and wives during pregnancy to prevent postpartum crises.

## 2 BACKGROUND

In Europe and the United States, the need for interventions to prevent the deterioration of marital relationships has been highlighted, and meta‐analyses have confirmed the effectiveness of educational programmes for couples (Pinquart & Teubert, 2010). In recent years, Japan has developed educational programmes for new parents to encourage fathers' participation in childcare and housework (Yamaguchi & Sato, 2016), programmes focusing on parenthood (Takeishi et al., 2019), and programmes to increase empathy among couples to prevent postpartum depression (Watanabe et al., 2019). In our previous studies, we developed a programme to enhance understanding of older primiparas' physical and mental health and to strengthen marital relationships (Nakajima et al., 2021). As a component of this programme, we aimed to strengthen couples' relationships by reinforcing three aspects of husbands' support for wives: mental support, housework support and communication to prepare for childcare. The previous study was a semi‐experimental study with no control group. We therefore considered it necessary to conduct a comparative study between the programme participants in that programme and non‐participant groups to determine whether the newly developed marital relationship enhancement programme for older primiparas is sufficiently versatile and effective. This study's research question is to evaluate and determine whether the programme promotes the stabilization of older primiparas' physical and mental health and strengthens marital relationships.

## 3 THE STUDY

### 3.1 Hypothesis

Hypothesis 1 Compared with the control group, the intervention group has a better understanding of the mental health of older primiparas, increased satisfaction among wives with the ‘mental support’ from their husbands and significantly greater reduction in wives' postpartum EPDS.

Hypothesis 2 Compared with the control group, the intervention group has a better understanding of the physical health of older primiparas, greater satisfaction among wives with ‘housework support’ and ‘childcare support’ from their husbands, and a significantly greater reduction in the wives' physical burden after childbirth.

Hypothesis 3 Compared with the control group, the intervention group shows significantly greater increases in wives' satisfaction with the three husbands' supports and much higher satisfaction with their postpartum marital relationship as reflected in the higher Quality Marriage Index (QMI).

### 3.2 Design

This study was a non‐randomized controlled trial that examined the programme's effects by allocating participants to two groups: an intervention group that participated in the programme to strengthen the relationship between husband and wife, and a control group that did not participate in the programme. A TREND checklist was completed (included as Appendix S1).

### 3.3 Research participants

Participants were older primiparas wives and their husbands who attended maternity classes at two obstetric hospitals in A prefecture. The intervention group was consisted of couples who participated in the programme in addition to the regular maternity class, while the control group consisted of couples who participated only in the normal maternity class. The content of both normal maternity classes at the two hospitals was designed to prepare the mind and body for childbirth, the delivery process, and signs of delivery. Thus, potential bias among study participants was minimized as their degree of knowledge and preparedness regarding childbirth during pregnancy were similar.

Participants were recruited through convenient sampling. The sample size was calculated using the power analysis software G *P power 3. Intervention effects were calculated at a significance level of α = 0.05, an effect size of 0.80 and a power of 0.80, the ideal sample size was 27 couples in the intervention group and the control groups, respectively.

Selection criteria for participants were as follows: (1) pregnant wives age ≥35 years, (2) couples who could participate in the study during pregnancy, as well as at 1 and 3 months after childbirth, and (3) couples who could speak and understand Japanese. The following were the exclusion criteria: (1) pregnant wives and their infants with serious health problems and (2) couples with a mental illness. The programme was implemented between April 2018 and August 2019.

### 3.4 Programme content

The programme was conducted with one group of two or three couples who were close to the pregnancy's full term. The programme was held on weekends so couples could participate together and took place once during pregnancy for duration of 2 h. The principal investigator and co‐principal investigator, a licenced midwife, conducted this programme with an older primiparous couple at a university. This programme was implemented to help couples understand the physical and mental risks faced by older primiparas and to strengthen the couple partnership (Nakajima et al., 2021).

The programme included the following: (1) greetings and self‐introductions (10 min); (2) information on the physical and mental risks of older primiparas and husbands' support (30 min); (3) couple discussion on postpartum couple relationships (15 min); (4) break (10 min); (5) interaction among participating couples (25 min); (6) couples' pair stretches (20 min), (7) completion of the programme evaluation questionnaire (10 min). The programme was implemented between April 2018 and August 2019.

### 3.5 Investigation procedure

The principal investigator explained the purpose of the study to the older primiparous wives who participated in a regular maternity class at the hospital. The husbands' cooperation was secured through their wives. The survey was conducted three times in total, that is during pregnancy, and at 1 and at 3 months after childbirth. The researchers distributed questionnaires and reply envelopes to each couple. The wives and husbands placed completed questionnaires in their respective envelopes following which the completed questionnaires were collected from each couple.

The data collection period was from April 2018 to December 2019.

### 3.6 Measurements

#### 3.6.1 Attributes

Participants responded to sociodemographic questions regarding their age, the establishment of the pregnancy, pregnancy progress, wives' employment, family structure and childcare situation (supporter/satogaeri; returning to their parent's' home).

#### 3.6.2 Quality Marriage Index

The QMI is a scale created by Norton (1983), translated into Japanese by Moroi (1996). It consists of six items (total score of 6 to 24) that question the quality of the marital relationship, with higher scores indicating greater marital satisfaction. Its reliability has been verified. Cronbach's alpha was 0.927. In this study, this scale assessed wives' and husbands' marital relationship satisfaction during pregnancy, and at 1 month and 3 months after childbirth.

#### 3.6.3 Wives' satisfaction with husbands' support

##### 3.6.3.1 Wives' satisfaction with husbands' support during pregnancy

The ‘scale of wives' satisfaction with husbands' supportiveness during pregnancy’ was developed by Nakajima and Tokiwa (2013). This scale is divided into two subscales, one for wives, and one for husbands and consists of three components: ‘wife's mental support’, ‘housework support’ and ‘couple's communication to prepare for childcare’. The higher the score, the more satisfied the wife is with her husband's support. The reliability and validity of the scale have been verified. Cronbach's alpha ranged from 0.83 to 0.86 for wives and husbands. We used this to assess wives' satisfaction with their husbands' support during pregnancy.

##### 3.6.3.2 Wives' satisfaction with husbands' support after childbirth

The ‘scale of wives' satisfaction with husbands' supportiveness after childbirth’ was used by Nakajima et al. (2016) with reference to the ‘husband's housework and childcare behavior scale’ developed by Hinokuma et al. (1998). This scale also has two subscales, one for wives and one for husbands. This scale consists of three components that measure ‘wife's mental support’, ‘housework support’ and ‘childcare support’. The higher the score, the more satisfied the wife is with the extent of her husband's support. In this study, wives' satisfaction with husbands' supportiveness was measured 1 month and 3 months after childbirth.

#### 3.6.4 Edinburgh Postnatal Depression Scale (EPDS)

The EPDS is a postpartum depression screening test developed by Cox et al. (1987). Okano created the Japanese version (Okano et al., 1996) and its reliability and validity have been verified. It consists of 10 items on a 4‐point Likert scale (0 to 30 points in total). Nine points or more are indicative of postpartum depression. Cronbach's alpha was 0.78. In this study, wives' EPDS was evaluated during pregnancy and at 1 month and 3 months after childbirth.

#### 3.6.5 Postpartum physical fatigue situation

The Fatigue Accumulation Checklist was developed by the Japan Industrial Safety and Health Association to self‐check the accumulation of workers' tiredness (Japan Industrial Safety and Health Association, 2004). It comprises two scales; one for the individual person and one for the family. Murakami (Murakami et al., 2008) used it to measure the physical fatigue of mothers after childbirth. The higher the score, the more physical fatigue experienced by the mother. In this study, we used the scale to measure the physical burden of older primiparas at 1 month and then again at 3 months after childbirth.

### 3.7 Method of analysis

Participant baseline characteristics and comparisons between the two groups were examined using the Mann–Whitney U *t*‐test and Pearson's chi‐square test. In evaluating the results, the Mann–Whitney U *t*‐test was applied for between‐group comparisons of the EPDS and QMI for the two groups. The Wilcoxon signed‐rank test was used for within‐group comparisons during pregnancy and at 1 month and 3 months after childbirth. ‘Wives' physical burden’ and ‘wives' satisfaction with husbands' support’ assessments were used to examined comparisons between the two groups, 1 month, and 3 months after delivery, between and within groups, by means of the paired *t*‐test. All analyses were performed using IBM SPSS Statistics 28.0. The significance level was set to at equal to or less than 0.05.

### 3.8 Ethical considerations

Participation in the study was voluntary. All participants were assured in writing and verbally that their refusal or exclusion from the study would not affect their healthcare services. Participants agreed that they had read and understood the terms and conditions and could withdraw from participation in the study at any time. The Clinical Research Ethical Review Board of [REDACTED] (approval number [REDACTED]).

## 4 RESULTS

### 4.1 Flow diagram of research participants

**FIGURE 1:**
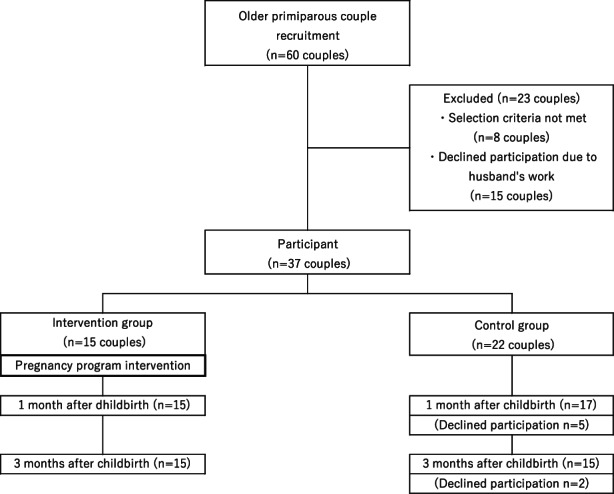
Flow diagram of research participants.

The researchers recruited 60 couples to participate in the study. After excluding couples who could not participate in the programme because of the husbands' work schedules, the 37 couples who met the selection criteria and agreed to participate in the study were finally assigned to one of the two groups. The intervention group consisted of 15 couples who had participated in the pregnancy programme. All intervention group participants responded to the questionnaires during pregnancy, 1 month and 3 months after delivery and submitted valid responses for analysis. The control group comprised 15 couples who had completed all the questionnaires during pregnancy, 1 month and 3 months after childbirth. A flow diagram of this study is shown in Figure 1. The data collection period was from April 2018 to December 2019.

### 4.2 Baseline characteristics of the participants and comparison between the two groups

The mean age of wives in the intervention group was 36.67 ± 1.63 years (range: 35–40 years), and the husbands' mean age was 38.00 ± 4.30 years (range: 32–45 years). In the control group, the wives had a mean age of 37.40 ± 2.01 years (range: 35–45 years), and their husbands' mean age was 37.67 ± 6.40 years (range: 28–55 years). There was no significant difference between the characteristics of the couples in the two groups, and there was also no significant difference in ‘wives' satisfaction with husbands' support’ in the participating intervention and control groups at the baseline level (Table 1). The mean number of gestational weeks for pregnant women was 31.5 ± 4.17 weeks (range 25–37) at the time of programme participation. The average number of couples participating in the programme was 2.5 per session.

**TABLE 1:** Baseline characteristics of the participants and comparison between the two groups.

	Intervention group (*n* = 15)	Control group (*n* = 15)	*p*‐value
**Age (years)**	**Mean ± SD (range)**		
Wife age (years)^a^	36.67 ± 1.63 (35–40)	37.40 ± 2.01 (35–45)	0.389
Husband age (years)^a^	38.00 ± 4.30 (32–45)	37.67 ± 6.40 (28–55)	0.870
**Background**	***n* (%)**		
Establishment of the pregnancy^b^			
Natural pregnancy	6 (20%)	7 (23.3%)	0.713
Infertility after pregnancy	9 (30%)	8 (26.7%)	
Pregnancy progress^b^			
Regular progress	15 (50%)	15 (50%)	1.000
Complication during pregnancy	0	0	
Wives' employment^b^			
Work	10 (37.3%)	10 (37.3%)	1.000
Housework	5 (16.7%)	5 (16.7%)	
Family structure^b^			
Nuclear family	12 (40%)	15 (50%)	0.112
Extended family	3 (3%)	0 (0%)	
Family support or satogaeri after childbirth^b^			
Yes	15 (50%)	14 (46.7%)	0.500
No	0 (0%)	1 (3.3%)	
**Wives' satisfaction with husbands’ support** ^a^	**Mean ± SD**		
Wives’ perception			
Wife's mental support^c^	29.87 ± 5.73	30.18 ± 5.59	0.869
Prepares for childcare^d^	32.2 ± 4.68	32.68 ± 5.73	0.790
Husband's perception			
Prepare for childcare^e^	25.07 ± 5.74	27.27 ± 4.41	0.195
Housework support^f^	10.87 ± 3.09	10.32 ± 3.94	0.654

^a^ Mann–Whitney U *t*‐test.

^b^ Pearson's chi‐square test.

^c^ Score range 40–8.

^d^ Score range 40–8.

^e^ Score range 35–7.

^f^ Score range 15–3.

### 4.3 Outcome evaluation

#### 4.3.1 Edinburgh Postnatal Depression Scale

The EPDS scores evaluating wives' mental health showed a significant decline in postpartum over time in the intervention group (pregnancy–3 months: *Z* = −2.297, *p* = 0.022, 1 month–3 months: *Z* = −2.285, *p* = 0.022; Table 2). However, comparisons between the two groups showed no significant differences.

**TABLE 2:**
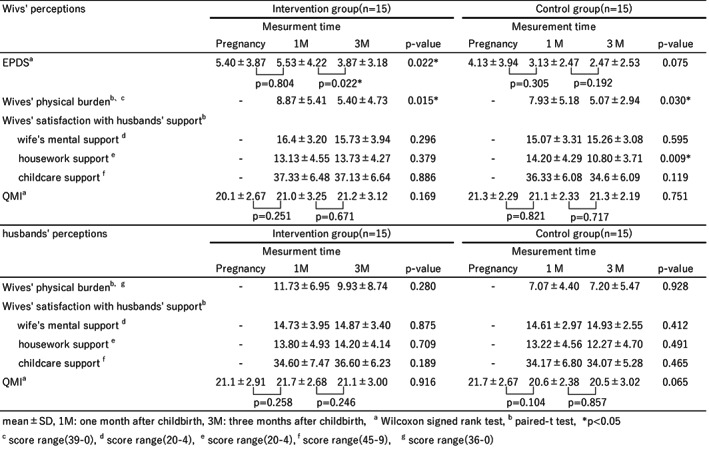
Physical and mental health, marital relationship changes from pregnancy to 1 and 3 months after childbirth.

#### 4.3.2 The physical burden

After participating in the programme, the physical burden on wives at 3 months was recorded as being significantly lower in the two groups at 3 months than at 1 month after childbirth in both groups (intervention group *t* = 2.767, *p* = 0.015; control group *t* = 2.408, *p* = 0.03; Table 2).

In addition, husbands' perceptions of their wives' physical burden were significantly higher in the intervention group (11.73) than in the control group (7.07) at 1 month postpartum (*t* = 2.197, *p* = 0.036).

#### 4.3.3 Wives' satisfaction with husbands' support

The ‘wife's mental support’ subscale showed no significant difference between the two groups in terms of change at 1 and 3 months postpartum for either wives or husbands. The ‘housework support’ subscale showed no significant difference between the two groups in the husbands' perceptions but was significantly lower in the wives' control group: 10.8 at 3 months postpartum compared to 14.2 at 1 month postpartum (*t* = 3.058, *p* = 0.009). The ‘childcare support’ subscale showed no significant difference between the two groups in terms of change at one and 3 months postpartum for either wives or husbands (Table 2).

### 4.4 Quality Marriage Index

The QMI, which measures marital relations, showed no significant difference between the two groups at 1 and 3 months postpartum for either the wives or the husbands (Table 2).

## 5 DISCUSSION

The study compared wives' satisfaction with mental support from their husbands after childbirth and found no significant differences between intervention and control groups. In addition, the wives' postpartum EPDS decreased with the passage of postpartum time in the intervention group, but comparisons between the two groups showed no significant differences. Thus, Hypothesis 1 was not supported.

There was no significant difference between the intra‐group comparison and the inter‐group comparison in the subscale ‘wife's mental support’. In other words, participation in the pregnancy programme did not affect the emotional bond between the couple. ‘Wife's mental support’ is expressed by the husband in words and attitudes that consider the wife (Nakajima & Tokiwa, 2013), which can make a difference in the perceptions of both the wife and the husband. In other words, couples in the intervention group who participated in the pregnancy programme and were able to understand their wives' mental health postpartum may have provided more mental support to their wives postpartum, but their wives may not have perceived it as satisfactory due to differences in their perceptions.

In addition, the EPDS, which was used to measure the mental health of wives, showed no significant decrease in the control group but decreased significantly over time in the intervention group. A meta‐analysis of perinatal depression in Japanese women shows that the prevalence of depression decreases over time in the postpartum period (Tokumitsu et al., 2020). Qualitative research by Nakajima et al. (2020) revealed that older primiparas were worried about pregnancy and childbirth but possessed significant mental and social strength. Thus, the older primiparas in this study were considered to have stable mental health throughout the postpartum course due to their participation in the programme.

Regarding the physical health of the older primiparas, husbands showed significantly greater understanding of their wives' physical burdens after childbirth in the intervention group than in the control group, and wives' showed significantly greater satisfaction with housework support from their husbands. Thus, Hypothesis 2 was supported.

Husbands' perceptions of their wives' physical burdens in the intervention group increased significantly 1 month after childbirth. In addition, ‘housework support’ on the subscale of ‘wife's satisfaction with husband's support’, was significantly reduced in the control group 3 months after childbirth. However, wives' perceptions in the intervention group did not decline, and no significant differences were found. In other words, husbands who participated in the pregnancy programme could understand the physical burden on their wives after childbirth thus avoiding a decrease in the wives' satisfaction with their husband's support. The husbands' housework support is a realistic supportive behaviour and is easily shared by the couple (Nakajima & Tokiwa, 2013). Therefore, it is suggested that this programme could strengthen the marital relationship because the husband's understanding of the wife's physical burden after childbirth improves the wife's satisfaction with her husband's support with the housework.

There were no significant differences in the QMI between the intervention and control groups regarding their satisfaction with the postnatal marital relationship. Therefore, Hypothesis 3 was not supported.

In this study, Hypothesis 1 regarding ‘wife's mental support’ was not supported while Hypothesis 2 regarding ‘wife's physical health and wife's household support’ was supported. There is also a correlation between ‘wife's mental support’ and QMI, and an increase in ‘wife's mental support’ leads to improved marital satisfaction (Nakajima et al., 2016). Therefore, the couple must understand their perception differences to enhance compassion from the husband towards the wife, and for the wife to appreciate the support of the husband. By strengthening the support for marital relationships in the pregnancy programme, it is expected that the satisfaction level of marital relationships will improve. By strengthening the ‘wives' mental support’ in the pregnancy programme as discussed above, it is expected that the satisfaction level of marital relationships will improve.

### 5.1 Limitations of the research and future issues

It was unfortunate that the couples who participated in the pregnancy programme reported no effect on the satisfaction of their marital relationship or the development of intimacy between them.

The following may have affected the results: (1) Since there are few general measurement tools that can be continued during pregnancy and after childbirth, a baseline measurement was not possible, therefore measurement was performed only after childbirth. (2) The use of convenience sampling. (3) The valid response rate was low because there was no cooperation between wives and husbands.

Based on the data obtained in this study, in the future, we intend to: (1) make the programme applicable, not only to older primipara couples but also to all primipara couples and (2) improve the pregnancy programme, and continue the development of the postnatal programme to promote intimacy between married couples following childbirth and (3) secure sufficient participants and verify the effects through randomized controlled trials.

## 6 CONCLUSION

This study evaluated a programme we developed for couples to enhance understanding of the physical and mental health of older primiparas and to strengthen marital relationships, by employing an intervention group and a control group. With respect to the evaluation results, there was a significant decrease in the EPDS scores for wives in the intervention group over the course of the postpartum period. However, there was no significant difference in the QMI scores for marital relationship satisfaction and the ‘wife's mental support’ subscale. However, participating in the pregnancy programme significantly increased husbands' awareness of their wives' physical burdens 1 month after childbirth. Additionally, the ‘housework support’ subscale 3 months after childbirth did not decline. It was suggested that this programme could possibly strengthen the marital relationship because the husband's enhanced understanding of the physical burden of the wife after childbirth leads to an improvement in the wife's satisfaction with the husband's support with housework.

## AUTHOR CONTRIBUTIONS

KN contributed to study design. KN and AH contributed to data analysis. KN and TN contributed to manuscript preparation.

## FUNDING INFORMATION

This research was supported by a Grant‐in‐Aid for Scientific Research in 2017 (Grant‐in‐Aid for Basic Research C).

## CONFLICT OF INTEREST STATEMENT

The authors have no potential conflicts of interest regarding this article's research.

## Supporting information


Appendix S1

## Data Availability

The data that support the findings of this study are available from the corresponding author upon reasonable request.
